# Prev-Sepse Pediátrica: content validation of a digital serious game on pediatric sepsis

**DOI:** 10.1590/0034-7167-2025-0099

**Published:** 2026-07-24

**Authors:** Jakeline Barbara Alves, Rosângela Aparecida Pimenta

**Affiliations:** I Universidade Estadual de Londrina. Londrina, Paraná, Brazil

**Keywords:** Sepsis, Pediatrics, Video Games, Education, Distance, Educational Technology., Sepsis, Pediatría, Juegos, Educación a Distancia, Tecnología Educacional.

## Abstract

**Objectives:**

to validate the content of a digital serious game on pediatric sepsis.

**Methods:**

methodological content validation study conducted in two rounds with expert judges (nurses and physicians). In the first round, the expert panel evaluated all screens of the initial prototype. After adjustments, the second version of the game underwent content validation using the Health Educational Content Validation Instrument (IVCES), and the agreement rate and Content Validity Index (CVI) were calculated.

**Results:**

in the first round, 11 judges evaluated the game and made suggestions, all of which were incorporated. The second round involved nine judges. The overall agreement rate for the prototype items was 100%, and the overall CVI was 0.99.

**Conclusions:**

the game was modified according to the judges’ feedback, and its content was validated for use as a tool for teaching and learning about pediatric sepsis.

## RESUMEN

**Objetivos:**

validar el contenido de un juego educativo digital sobre sepsis pediátrica.

**Métodos:**

estudio metodológico de validación de contenido realizado en dos rondas con jueces expertos (enfermeros y médicos). En la primera ronda, los jueces evaluaron todas las pantallas de la versión inicial del prototipo. Tras los ajustes, la segunda versión del juego se sometió a validación de contenido mediante el Instrumento de Validación de Contenido Educativo en Salud, y se calcularon el porcentaje global de acuerdo y el índice de validez de contenido (IVC).

**Resultados:**

en la primera ronda, 11 jueces realizaron la evaluación y emitieron sugerencias, que fueron incorporadas. En la segunda ronda participaron nueve jueces. El porcentaje global de acuerdo fue del 100% y el IVC global fue de 0,99.

**Conclusiones:**

el juego fue modificado de acuerdo con las consideraciones de los jueces y su contenido quedó validado como herramienta de enseñanza-aprendizaje sobre sepsis pediátrica.

## INTRODUCTION

The postmodern era, characterized by globalization and technological advances, has led education systems to adopt emerging teaching methods, particularly in health care and health education. Information and communication technologies (ICT) represent an innovative teaching and learning strategy; however, given the multiple ways information is disseminated, the content shared is often inaccurate or poorly aligned with scientific evidence. In this context, games designed for educational purposes are known as “serious games” or “learning games^(1-3)^.

Games are commonly used for entertainment; however, in serious games, educational content and enjoyment must be balanced so that neither predominates. Digital games have the advantage of being easily accessible, as they can be used anytime, anywhere on mobile devices^(4)^.

In health education, technologies are viewed as important resources for teaching students and professionals and for promoting health in the general population^(5-7)^. Continuing education and in-service training in health services are responsible for keeping professionals up to date and improving their skills, as well as those of students in teaching hospitals^(8)^. The literature shows progress in the use of technologies as a teaching strategy, although this development is more evident in basic and higher education. In clinical practice, health professionals can use games and applications to support patient care, facilitate problem identification, and assist in diagnostic reasoning.

In health care, sepsis is a major condition with serious consequences and is considered a public health problem because it increases mortality, length of hospital stay, and hospital costs, especially in the pediatric population^(9)^. In a scoping review of educational technologies on sepsis, only three of nine studies reported using games, and only one focused on the pediatric age group, underscoring how limited educational initiatives on pediatric sepsis for students and health professionals still are^(10-15)^.

Given the impact of pediatric sepsis on children’s health and the scarcity of games and applications for education on this topic, a digital serious game on pediatric sepsis was developed. In this context, validating the content conveyed by such technologies is essential to ensure high quality material grounded in scientific evidence^(16)^.

### Study relevance

This article derives from the doctoral dissertation “*Prev-sepse pediátrica: criação e validação de um jogo educativo digital para estudantes e profissionais*”^(10)^, deposited in the institutional repository and available at: https://repositorio.uel.br/srv-c0003-s01/api/core/bitstreams/bb57156c-e31b-4a4b-bc4a-d0266f152a5e/content. It aims to contribute to the professional development of healthcare professionals regarding pediatric sepsis through a digital teaching and learning strategy.

## OBJECTIVES

To validate the content of a digital serious game on pediatric sepsis for students and health professionals.

## METHODS

### Ethical aspects:

This study is part of the research project “Evaluation of healthcare-associated infections in children and adolescents,” approved by the Research Ethics Committee of the State University of Londrina under CAAE 28068119600005231. Before accessing the evaluation and validation forms for the prototype, the judges were invited to read and voluntarily agree to the Informed Consent Form (ICF).

### Type of study

This methodological study aimed to validate the content of the digital serious game “*Prev Sepse Pediátrica*”, for which a trademark registration application has been filed with the Brazilian National Institute of Industrial Property (INPI) under number 934465100.

### Theoretical-methodological framework

The game was developed using a design thinking approach, which comprises the stages of inspiration, ideation, and implementation; the creative process is described in detail in a separate publication on the game development process^(10)^.

### Methodological procedures

In the inspiration stage, users’ needs are explored, and the key problems and core game content are defined. To this end, we conducted a scoping review that identified a small number of studies on pediatric sepsis education using educational technologies. In the ideation stage, ideas are generated to address the identified problem and to design the technology-in this case, the prototype of the digital serious game on pediatric sepsis^(17,18)^. In the implementation stage, user testing is carried out in two rounds: the first is considered a pilot, and the second is the validation round, which is the focus of the present study. This two-stage design reflects the iterative nature of design thinking^(18)^.

The game is structured in five phases. Phase 1 covers basic, essential concepts; Phase 2 guides players in identifying suspected sepsis based on signs of the inflammatory response; Phase 3 addresses signs of organ dysfunction; Phase 4 focuses on initial management in suspected or confirmed sepsis; and Phase 5 addresses the prevention of healthcare-associated infections^(19-23)^.

### Data source

For the pilot evaluation, 19 judges were recruited through purposive, non-probability sampling. Eligibility criteria included holding a degree in Nursing or Medicine, having at least one graduate certificate in pediatrics, and working in pediatric care in secondary or tertiary services in Londrina, Paraná State, Brazil. The judges were contacted by the first author via e-mail, and 11 completed an online survey using Google Forms between December 2023 and January 2024^(24)^.

The content of the game screens was developed using material from national and international manuals and protocols on healthcare-associated infections (HAIs) and pediatric sepsis, including concepts, signs and symptoms, initial management, and prevention^(19-23)^.

The form included all screens of the prototype. For each content screen, there was a corresponding question addressing the specific information presented, totaling 50 questions, with responses given on a five-point Likert-type scale: 5) strongly agree; 4) somewhat agree; 3) neither agree nor disagree; 2) somewhat disagree; and 1) strongly disagree. An additional open-ended question allowed judges to provide suggestions or comments on each screen.

After the first round and the subsequent adjustments based on the judges’ suggestions, the 11 judges were invited to participate in the second round, in which they completed the content validation form.

From May to July 2024, this form was available via Google Forms and included items on the judges’ characteristics, the game name, the prototype screens, and an open-ended question for additional comments. After evaluating all screens, the judges rated the game using the Health Educational Content Validation Instrument (IVCES), previously validated by Leite *et al.* ^(25)^.

The IVCES comprises 18 items distributed across three domains: Objectives, which address the purposes and goals of the content; Structure/presentation, which includes organization, structure, strategy, coherence, and sufficiency; and Relevance, which considers the content’s significance, impact, and its ability to motivate and sustain interest^(25)^.

At this stage, the judges selected the option that best reflected their opinion on each item in the domains described in detail in Figure 1, choosing from the following alternatives: Disagree (0), Partially agree (1), and Totally agree (2).

### Data analysis

The open-ended comments were analyzed in full, and the quantitative items were summarized using the overall agreement rate and the Content Validity Index (CVI) for each item. The agreement rate was calculated by summing the number of responses scored as 1 or 2 and dividing this value by the total number of ratings. For each item, the CVI was obtained by dividing the number of responses scored as 1 or 2 by the total number of responses for that item. Items were considered valid when agreement exceeded 80% and the CVI was > 0.80^(25,26)^.

## RESULTS

The pilot evaluation involved 11 judges, 5 nurses, and 6 physicians. Regarding academic qualifications, one had completed postdoctoral training, four held doctoral degrees, three held master’s degrees, and three held graduate certificates. Nine judges completed the content validation form (Table 1).

**Table 1:** Characteristics of the judges in the second round of game evaluation according to profession, years since graduation, specialty, years of professional experience, and highest academic degree, Londrina, Paraná, Brazil, 2024

Judge	Profession	Years since graduation	Specialty	Years of professional experience	Highest academic degree
1	Nurse	27	Pediatrics	27	Doctoral degree
2	Nurse	17	Pediatrics	17	Postdoctoral training
3	Nurse	15	Pediatrics	15	Doctoral degree
4	Physician	23	Pediatric intensive care	20	Doctoral degree
5	Physician	30	Pediatric infectious diseases	27	Doctoral degree
6	Physician	40	Pediatric intensive care	32	Master’s degree
7	Physician	27	Pediatric infectious diseases	24	Master’s degree
8	Nurse	18	Pediatrics and infectious diseases	10	Master’s degree
9	Physician	16	Pediatrics	7	Graduate certificate

The judges’ suggestions in the first round regarding vital signs, healthcare-associated infections (HAIs), and initial management were incorporated. Reference values for vital signs were organized in a table, and only laboratory tests with reference ranges reported in national or international guidelines were included. Literature citations, enabling access to the source material, were also added to the screens whenever deemed necessary. Suggestions concerning the concepts of systemic inflammatory response syndrome (SIRS), severe sepsis, and septic shock were only partially incorporated.

While this prototype was being evaluated and validated, new international guidelines for the diagnosis and management of pediatric sepsis were published. The concept and definitions of sepsis were updated in line with the judges’ suggestions; however, the signs of systemic inflammatory response were retained because one of the aims of the game is to support early identification by strengthening knowledge of these signs^(20)^.

Furthermore, as the new guidelines define sepsis in terms of organ dysfunction, the game may help identify signs that precede severe clinical deterioration. In addition, as of the time of this validation, no national guideline had been published establishing sepsis criteria specific to the Brazilian pediatric population^(19-21)^.

Table 1 describes the judges’ characteristics by profession, years since graduation, specialty, years of professional experience, and highest academic degree.

After this characterization, the judges completed the validation form adapted from the IVCES. All comments and qualitative remarks were analyzed in full.

Regarding the game title, *Prev-Sepse Pediátrica*, all judges agreed with it and did not suggest any changes or improvements.

As in the pilot evaluation, the updated sepsis management guidelines (Phoenix criteria) were also mentioned in the validation stage. These criteria are based on grading the degree of organ dysfunction and do not provide detailed descriptions of early signs of sepsis that can be identified by any health professional, even in the absence of laboratory results or more comprehensive assessments of renal perfusion or respiratory function. For this reason, suggestions related to the new criteria were accepted only to update the definitions of sepsis and septic shock^(19-21)^.

The fever threshold was also discussed. Although an axillary temperature above 37.8 °C is usually considered fever, given the requirement for up-to-date, evidence-based content, the reference temperature reported in the literature for pediatric sepsis cases was adopted^(19,27)^.

Regarding the request for a more recent reference from the Latin American Sepsis Institute, no new recommendations had been issued by the time of this study. However, when revising the prototype, the authors considered the recent publication of international criteria for sepsis^(19-21)^, which informed some of the suggestions incorporated into the game.

One judge suggested adding fluid balance calculations, but it was decided to retain only the underlying concept. For capillary refill time (CRT), another judge proposed including markedly shortened CRT as a diagnostic criterion for distributive shock, but the original description was also maintained. This decision reflected the fact that current sepsis diagnostic recommendations address only prolonged CRT, and the aim was to align the content with the selected references; in addition, only one judge requested this change.

The suggestions to present healthcare-associated infections (HAIs) as the main infection types and to add removal of the endotracheal tube on the previous day as a diagnostic criterion for ventilator-associated pneumonia were accepted.

The overall agreement rate for the items-considering the responses “Partially agree” and “Totally agree”-was 100%, because no item received a “Disagree” response.

Regarding the CVI for each item, the results of the calculations are presented in Table 2, which describes the prototype of the digital serious game Prev-Sepse Pediátrica. Most CVI values exceeded 0.90, yielding an overall CVI of 0.99, which was considered adequate^(24-26,28)^.

**Table 2:** Content Validity Index (CVI) per item, according to the Health Educational Content Validation Instrument, for evaluating the prototype of the digital serious game *Prev-Sepse Pediátrica*, Londrina, Paraná, Brazil, 2024

Domains and items	CVI^*^
**OBJECTIVES: purposes and goals of the content**	
1. Covers the proposed topic	1.00
2. Is appropriate for teaching and learning	0.94
3. Clarifies questions about the topic	1.00
4. Promotes reflection on the topic	1.00
5. Encourages behavior change	1.00
**STRUCTURE/PRESENTATION: organization, structure, strategy, coherence, and sufficiency**	
6. Uses language appropriate for the target audience	1.00
7. Uses language appropriate for this educational material	1.00
8. Uses interactive language that enables active involvement in the educational process	1.00
9. Provides accurate information	0.88
10. Provides objective information	1.00
11. Provides clear information	1.00
12. Provides the necessary information	1.00
13. Presents ideas in a logical sequence	1.00
14. Addresses a current topic	1.00
15. Has an appropriate length	1.00
**RELEVANCE: significance, impact, motivation, and interest**	
16. Promotes learning	1.00
17. Contributes to knowledge in the field	
18. Stimulates interest in the topic	1.00
Total	0.99

* *CVI - Content Validity Index.*

*Source: Adapted from Leite et al. (2018).*

## DISCUSSION

Content validation of an educational technology plays a central pedagogical role in a context characterized by multiple channels for disseminating information, where content often circulates without scientific support and may mislead individuals. This situation is particularly concerning for both initial training and continuing professional education, especially in health care, where decisions based on inadequate information can compromise patient safety^(2,16)^.

The IVCES used in this study was developed to support the validation of educational materials for different target audiences while preserving key instructional features. Its items were developed based on instructional design principles, which systematically guide the planning and development of educational materials for various teaching modalities (face-to-face, blended, and distance learning). Given these characteristics, the instrument is appropriate for validating the prototype developed in this study^(25)^. Similarly, a study that validated an educational technology on sepsis in adults used the IVCES to assess its content and collected responses via an electronic form created in Google Forms^(29)^.

Few studies have been identified that validate the content of sepsis-related educational technologies. Among this small group, Limeira *et al.* (2023) validated an app on sepsis in adults aimed at educating the general population^(29)^. Evans *et al.* (2015) and Adhikari *et al.* (2021) developed games on sepsis in adults, neither study reported content validation; instead, both evaluated only the games’ impact on students’ knowledge^(12,14)^.

A simple search of the literature shows that most available studies are not directed toward educating health professionals, despite the increasing adoption of distance education^(30)^. Moraes *et al.* (2022) emphasize the use of educational games in continuing professional education and point to the need for more games focused on education about healthcare-associated infections^(31)^. Marotta and Mello (2019) state that classroom teaching requires dynamism and interactivity and that teachers need to adapt to new teaching methods and use them as effectively as possible; one strategy is to develop educational software tailored to specific areas of interest^(2)^.

One of the main principles in developing a learning game is to clearly define both the game goal and the learning objective. According to the judges’ assessments, the prototype in this study met this requirement. A game can only be considered a serious game when it is tied to a specific objective while still entertaining the player; in this way, it can not only promote knowledge but also foster behavior change^(4,31,32)^.

In a serious game, content should always be grounded in up-to-date scientific evidence because the material is intended to support players’ academic training and future professional practice. Accordingly, the references used in this prototype were the most recent publications from national and international organizations^(19-21)^. This attention to keeping the evidence current is also seen in other studies: Limeira *et al.* used similar references in their app on sepsis in adults, as did Evans *et al.* (2015), both consistent with the period in which each technology was developed and published^(14,29)^.

The prototype was structured into five phases. This stepwise instructional plan-whose logical sequence mirrors the sequence of steps involved in managing pediatric sepsis-supports learning and prepares players to act in real cases^(4,33)^.

The language was designed to be appropriate for both students and professionals while preserving the technical terminology of the healthcare field, helping keep players engaged through an attractive game experience^(25,33)^.

Other studies validating educational technologies have also used the CVI, which is recommended for assessing the content of educational materials^(29,34)^. The CVI values observed in this study were similar to or higher than those reported in other investigations. This finding may be related to the evaluation method adopted for the prototype, which followed a design thinking approach. The first analysis, conducted item by item on the pilot prototype screens, allowed for a more detailed assessment. The second analysis, in turn, focused on evaluating the game phases and used a specific instrument for educational content validation, providing a more conclusive assessment of the process. An additional advantage was that the judges were the same professionals who had previously interacted with the pilot, which gave them greater familiarity with the material and made content evaluation easier and faster^(18,34,35)^.

Furthermore, this prototype was designed in line with recommendations for developing learning games, which address most of the items evaluated by the instrument. These recommendations include making the learning objective clear from the outset of the game; using simple, straightforward language; incorporating visual resources and terminology specific to the healthcare field; and focusing on key priorities, such as identifying signs and symptoms of sepsis in children, implementing immediate actions once sepsis is suspected, determining the underlying causes, and preventing HAIs^(4,25,33,36)^.

In validating a game on surgical infection prevention, Nascimento (2021) used a script based strategy for content validation. The author chose to validate the content in its in-game format to make it easier for judges to understand how the material would be presented to users^(35)^.

It is important to integrate prototype development and content validation into the software development process, since the prototype serves as a guide for the judges and helps save time and avoid additional material costs^(22)^.

### Study limitations

Among the limitations of this study, one is the use of a purposive, non-probability sampling to recruit the judges; another is that the content was validated for use only with students and health professionals affiliated with the study setting. As a result, potential cultural differences in other regions where the material may be used were not considered, even though the content was developed in accordance with national and international consensus documents.

### Contributions to the Field

The validation performed by this study contributes to the dissemination of accurate and responsible information about pediatric sepsis to students and healthcare professionals.

## CONCLUSIONS

The overall CVI of 0.99 indicates that the content of this digital serious game addresses the intended learning objectives, has a structure and presentation that support learning, and is relevant for current teaching practice. Now that the content has been validated, the next step is to conduct usability testing by implementing the game with the target audience to assess its effectiveness as a teaching and learning strategy for pediatric sepsis.

## Data Availability

The research data are available within the article.
