# Supplementary material for: Prev-Sepse Pediátrica: content validation of a digital serious game on pediatric sepsis
Source: Rev Bras Enferm. 2026 Jul 24;79:e20250099. doi: 10.1590/0034-7167-2025-0099 (PMC13403641; doi:10.1590/0034-7167-2025-0099)
Supplement: Translation

# Translation: Prev-Sepse Pediátrica: validação de conteúdo de um jogo educativo digital

**DOI:** 10.1590/0034-7167-2025-0099pt

**Authors:** Jakeline Barbara Alves, Rosângela Aparecida Pimenta

**Affiliations:** I Universidade Estadual de Londrina. Londrina, Paraná, Brasil

**Keywords:** Sepse, Pediatria, Jogos, Educação *Online*, Tecnologia Educacional.

## Abstract

**Objetivos:**

validar o conteúdo de um jogo educativo digital sobre sepse pediátrica.

**Métodos:**

estudo metodológico para a validação de um jogo, realizada em duas rodadas por juízes (enfermeiros e médicos). Na primeira, eles avaliaram todas as telas da primeira versão do protótipo. Após ajustes, a segunda versão do jogo foi enviada para a validação do conteúdo utilizando-se o Instrumento de Validação de Conteúdo Educativo em Saúde, com cálculo do percentual de concordância e Índice de Validade de Conteúdo (IVC).

**Resultados:**

na primeira rodada, 11 juízes realizaram a avaliação e emitiram sugestões, que foram acatadas. A segunda rodada envolveu nove juízes. O percentual de concordância geral para os itens do protótipo foi de 100%, e o IVC geral dos itens foi 0,99.

**Conclusões:**

o jogo foi modificado conforme considerações dos juízes; e seu conteúdo, validado para ensino e aprendizagem sobre sepse pediátrica.

## INTRODUÇÃO

A era pós-moderna, caracterizada pela globalização e avanço da tecnologia, leva as redes de educação a integrarem-se nos métodos emergentes de ensino, sobretudo na área da saúde. As tecnologias de informação e comunicação são uma estratégia de ensino e aprendizagem inovadora; no entanto, diante das múltiplas formas de disseminar a informação, muitas vezes o conteúdo divulgado é incorreto ou pouco alinhado às evidências científicas. Nesse cenário, os jogos com proposta educativa são chamados “jogos sérios” ou “jogos de aprendizagem”^(1-3)^.

Os jogos são comumente utilizados para o entretenimento; porém, em se tratando de jogos sérios, o conteúdo e a diversão devem ser balanceados de maneira que um não sobreponha o outro. Os jogos digitais têm a vantagem de permitir maior facilidade de acesso, já que podem ser utilizados em qualquer hora e lugar, por meio de dispositivos móveis^(4)^.

No tocante à educação em saúde, as tecnologias são consideradas um apoio no ensino de estudantes e profissionais e na promoção da saúde da população geral^(5-7)^. A educação continuada e permanente nos serviços é responsável pela atualização e aprimoramento dos profissionais, bem como dos estudantes quando se trata de hospitais vinculados a redes de ensino^(8)^. Percebe-se, na literatura, avanço no uso das tecnologias como estratégia de ensino, porém esse progresso se destaca na educação básica e superior. Cabe aos profissionais da saúde o uso de jogos e aplicativos para fins de atendimento ao usuário, facilidade na identificação de problemas e auxílio em diagnósticos.

No âmbito da assistência à saúde, um dos agravos de grande repercussão é a sepse, considerada problema de saúde pública pelo aumento da mortalidade, tempo de internação e custos hospitalares, principalmente na população pediátrica^(9)^. Em revisão de escopo sobre as tecnologias educativas aplicadas ao conteúdo sobre sepse, identificou-se que, de nove estudos, somente três referiram o uso de jogos propriamente ditos, e apenas um abordou a faixa etária pediátrica, indicando a fragilidade das iniciativas educativas voltadas a estudantes e profissionais sobre sepse em crianças^(10-15)^.

Diante do impacto da sepse pediátrica sobre a saúde das crianças e da escassez de jogos e/ou aplicativos para educação sobre o tema, foi desenvolvido um jogo educativo digital sobre sepse pediátrica. Nesse sentido, a validação do conteúdo veiculado por essas tecnologias é necessária para assegurar um material de qualidade, fundamentado em evidências científicas^(16)^.

### Relevância do estudo

Este artigo é produto da tese “Prev-sepse pediátrica: criação e validação de um jogo educativo digital para estudantes e profissionais”^(10)^, depositada no repositório institucional e acessível pelo seguinte link: https://repositorio.uel.br/srv-c0003-s01/api/core/bitstreams/bb57156c-e31b-4a4b-bc4a-d0266f152a5e/content. Busca contribuir para a atualização de profissionais de saúde no que diz respeito à sepse pediátrica por meio de uma estratégia de ensino aprendizagem digital.

## OBJETIVOS

Validar o conteúdo de um jogo educativo digital sobre sepse pediátrica para estudantes e profissionais da saúde.

## MÉTODOS

### Aspectos éticos

Este estudo faz parte do projeto de pesquisa intitulado “Avaliação das infecções relacionadas à assistência à saúde em crianças e adolescentes”, aprovado pelo Comitê de Ética em Pesquisa da Universidade Estadual de Londrina sob o CAAE 28068119600005231. Antes de terem acesso aos formulários de avaliação e validação do protótipo, os juízes foram convidados a ler e aceitar, de forma voluntária, o Termo de Consentimento Livre e Esclarecido (TCLE).

### Tipo de estudo

Trata-se de uma pesquisa metodológica para validar o conteúdo do jogo educativo e digital “Prev-Sepse Pediátrica”, que possui solicitação de registro de marca no Instituto Nacional de Proteção Intelectual (INPI), sob o número 934465100.

### Referencial teórico-metodológico

O jogo foi desenvolvido conforme a metodologia de *design thinking*, que segue as etapas de inspiração, ideação e implementação; o detalhamento do processo criativo é explanado em estudo próprio de criação^(10)^.

### Procedimentos metodológicos

Na etapa de inspiração, recolhem-se as necessidades dos indivíduos, bem como definem-se os problemas e o conteúdo essencial para o jogo. Para tanto, foi realizada uma revisão de escopo, que identificou um baixo número de estudos abordando a educação com uso de tecnologias sobre sepse em crianças. Na etapa da ideação, surgem as ideias para a resolução do problema e criação da tecnologia - no nosso caso, a criação do protótipo do jogo educativo e digital sobre sepse pediátrica^(17,18)^. Na fase de implementação, são realizados testes com usuários, conduzidos em duas rodadas: a primeira é considerada um piloto; e a segunda é a rodada de validação, correspondente ao presente estudo. Esse delineamento em duas etapas corrobora a característica iterativa do *design thinking* ^(18)^.

O jogo propõe um esquema de fases. Fase 1 - conceitos básicos e indispensáveis; Fase 2 - instrução sobre como identificar os casos suspeitos de sepse com base nos sinais de resposta inflamatória; Fase 3 - sinais de disfunção orgânica; Fase 4 - aprendizado das primeiras condutas diante de caso suspeito ou confirmado de sepse; e Fase 5 - orientação sobre como prevenir infecções relacionadas à assistência à saúde^(19-23)^.

### Fonte de dados

Para a avaliação do piloto, foram convidados 19 juízes. A escolha deles foi feita de forma intencional não probabilística, seguindo os critérios de ser graduado em Enfermagem ou Medicina, ter no mínimo uma pós-graduação em pediatria e atuar na área em serviços de atenção secundária ou terciária no município de Londrina, estado do Paraná (PR), Brasil. Os juízes foram contatados pela autora via e-mail, dos quais 11 responderam um formulário de pesquisa via *Google Forms* entre dezembro de 2023 e janeiro de 2024^(24)^.

O conteúdo das telas do jogo foi desenvolvido de acordo com o material disponível em manuais e protocolos nacionais e internacionais sobre infecção relacionada à assistência à saúde (IRAS) e sepse pediátrica, que abordam conceitos, sinais e sintomas, primeiras condutas e prevenção^(19-23)^.

O formulário continha todas as telas do protótipo do jogo. Junto com cada tela de conteúdo, havia uma questão sobre a especificidade disponível na tela, totalizando 50 questões com alternativas de resposta do tipo likert de cinco pontos: 5) concordo muito; 4) concordo pouco; 3) não concordo, nem discordo; 2) discordo pouco; 1) discordo muito. Também constava uma questão aberta para o avaliador dar sugestões ou fazer comentários sobre cada tela.

Concluída a primeira rodada com os ajustes decorrentes das sugestões dos avaliadores, os 11 juízes foram convidados para a segunda rodada, na qual responderam ao formulário de validação de conteúdo.

De maio a julho de 2024, o formulário foi disponibilizado via Google Forms, com questões sobre a caracterização do juiz, nome do jogo, telas do protótipo e uma questão aberta para considerações adicionais. Após avaliar todas as telas, os juízes escolheram as alternativas de acordo com o Instrumento de Validação de Conteúdo Educativo em Saúde (IVCES), previamente validado por Leite *et al.* ^(25)^.

O IVCES é um instrumento que contém 18 itens, divididos em três domínios: Objetivos relacionados aos propósitos, metas ou finalidades do conteúdo; Estrutura/apresentação, que inclui organização, estrutura, estratégia, coerência e suficiência; e Relevância, que trata da significância, impacto, motivação e interesse pelo conteúdo^(25)^.

Diante disso, os juízes selecionaram a opção que mais se adequasse à sua opinião sobre os itens de cada domínio descritos detalhadamente na Figura 1, dentre as alternativas Discordo (0), Concordo parcialmente (1) e Concordo totalmente (2).

### Análise dos dados

As considerações foram analisadas na íntegra, e as questões alternativas quantitativas foram contabilizadas por meio do cálculo de percentual de concordância geral e Índice de Validade de Conteúdo (IVC) por item. Foi calculado o percentual de concordância por meio da soma do número de respostas 1 ou 2, dividida pelo número total de avaliações. Para cada item, o IVC foi obtido dividindo-se o número de respostas 1 ou 2 pelo número total de respostas do item. Considerou-se o item válido quando apresentou concordância > 80% e IVC > 0,80^(25,26)^.

## RESULTADOS

A avaliação do piloto do jogo foi realizada por 11 juízes, entre os quais 5 enfermeiros e 6 médicos. Quanto à titulação acadêmica, havia um pós-doutor, quatro doutores, três mestres e três especialistas. Nove responderam o formulário de validação de conteúdo (Tabela 1).

**Tabela 1:** Caracterização dos juízes da segunda rodada de avaliação do jogo quanto à profissão, tempo de formação, especialidade, tempo de experiência na área e maior titulação, Londrina, Paraná, Brasil, 2024

Juiz	Profissão	Tempo de formação (em anos)	Especialidade	Tempo de experiencia na área (em anos)	Maior titulação
1	Enfermeiro	27	Pediatria	27	Doutorado
2	Enfermeiro	17	Pediatria	17	Pós-doutorado
3	Enfermeiro	15	Pediatria	15	Doutorado
4	Médico	23	Medicina intensiva pediátrica	20	Doutorado
5	Médico	30	Infectologia pediátrica	27	Doutorado
6	Médico	40	Medicina intensiva pediátrica	32	Mestrado
7	Médico	27	Infectologia pediátrica	24	Mestrado
8	Enfermeiro	18	Pediatria e infectologia	10	Mestrado
9	Médico	16	Pediatria	7	Especialista

As considerações propostas pelos juízes na primeira rodada, referentes aos sinais vitais, IRAS e primeiras condutas, foram acatadas. Os valores de referência dos sinais vitais foram tabelados e, para os exames laboratoriais, foram acrescentados apenas aqueles cujos valores de referência constavam em diretrizes nacionais ou internacionais. As citações da literatura, com a possibilidade de acesso ao material, também foram incluídas nas telas sempre que julgadas necessárias. Com relação aos conceitos de síndrome da resposta inflamatória sistêmica (SIRS), sepse grave e choque séptico, as sugestões foram acatadas parcialmente.

No período de avaliação e validação deste protótipo, foram publicadas as novas diretrizes internacionais de diagnóstico e tratamento de sepse pediátrica. O conceito e as definições de sepse foram modificados conforme sugestões dos juízes, mas os sinais de resposta inflamatória sistêmica foram mantidos, já que um dos objetivos do jogo é auxiliar na identificação precoce por meio do conhecimento dos sinais de resposta inflamatória sistêmica^(20)^.

Ademais, as novas diretrizes estabelecem a sepse como uma disfunção orgânica, portanto o jogo pode ser útil para identificar os sinais que possam aparecer antes da instalação de um quadro grave, além de não haver, até a data desta validação, uma diretriz nacional que estabelece critérios válidos para a população brasileira^(19-21)^.

A Tabela 1 descreve a caracterização dos juízes de acordo com profissão, tempo de formação, especialidade, tempo de experiência na área e titulações.

Após a caracterização, eles responderam o formulário de validação adaptado do IVCES. Os comentários e considerações foram analisados na íntegra.

Com relação ao nome do jogo, Prev-Sepse Pediátrica, todos os juízes concordaram e não deram sugestões nem fizeram comentários para mudanças e aprimoramento.

Assim como na avaliação do projeto-piloto, também foram citadas na etapa de validação as novas diretrizes de atendimento da sepse (critérios de Phoenix). Estas se baseiam na identificação do grau de disfunção orgânica e não contemplam, de forma detalhada, os sinais precoces de sepse que podem ser identificados por qualquer profissional de saúde, mesmo na ausência de resultados de exames ou de uma avaliação mais completa da perfusão renal ou da função respiratória, por exemplo. Por esse motivo, as sugestões relativas aos novos critérios foram aceitas, mas apenas no que diz respeito às definições de sepse e de choque séptico^(19-21)^.

Foram feitas considerações sobre o limite da febre. Normalmente, considera-se febre uma temperatura axilar acima de 37,8 ºC, no entanto, dada a exigência de conteúdo apoiado em evidências científicas, foi adotada a temperatura de referência encontrada na literatura para os casos de sepse em pediatria^(19,27)^.

Sobre o questionamento acerca de uma referência mais atualizada do Instituto Latino-Americano de Sepse, não foram publicadas novas recomendações até o momento desta pesquisa. Entretanto, considerou-se, na revisão do protótipo, a publicação recente de novos critérios internacionais para sepse^(19-21)^, que subsidiaram parte das sugestões incorporadas.

Um juiz sugeriu acrescentar o cálculo de balanço hídrico, mas optou-se em manter apenas o conceito. Sobre a avaliação do Tempo de Enchimento Capilar (TEC), em que um juiz sugeriu acrescentar o TEC muito rápido como critério diagnóstico de choque distributivo, também foi mantida a descrição. Isso porque as recomendações para diagnóstico de sepse tratam apenas do TEC prolongado, a fim de alinhar o conteúdo às referências utilizadas, e porque apenas um juiz solicitou essa inclusão.

Foram acatadas as sugestões sobre colocar as IRAS como as principais infecções e acrescentar a retirada do tubo orotraqueal no dia anterior como critério diagnóstico para Pneumonia Associada à Ventilação Mecânica.

O percentual de concordância geral dos itens (Concordo parcialmente e Concordo totalmente) entre os juízes foi igual a 100%, porque não houve discordância total em nenhum item.

No tocante ao IVC por item, o resultado de cada cálculo está demonstrado na Tabela 2, que caracteriza o protótipo do jogo educativo digital Prev-Sepse Pediátrica. A maioria dos resultados do IVC foram superiores a 0,90, resultando em um IVC total igual a 0,99, considerado adequado^(24-26,28)^.

**Tabela 2:** Índice de Validade de Conteúdo por item, conforme Instrumento de Validação de Conteúdo Educativo em Saúde, quanto à avaliação do protótipo do jogo educativo digital Prev-Sepse Pediátrica, Londrina, Paraná, Brasil, 2024

Domínios e itens	IVC^*^
**OBJETIVOS: propósitos, metas ou finalidades**	
1. Contempla tema proposto	1,00
2. É adequado ao processo de ensino-aprendizagem	0,94
3. Esclarece dúvidas sobre o tema abordado	1,00
4. Proporciona reflexão sobre o tema	1,00
5. Incentiva mudança de comportamento	1,00
**ESTRUTURA/APRESENTAÇÃO: organização, estrutura, estratégia, coerência e suficiência**	
6. Usa linguagem adequada ao público-alvo	1,00
7. Usa linguagem apropriada ao material educativo	1,00
8. Usa linguagem interativa, permitindo envolvimento ativo no processo educativo	1,00
9. Fornece informações corretas	0,88
10. Fornece informações objetivas	1,00
11. Fornece informações esclarecedoras	1,00
12. Fornece informações necessárias	1,00
13. Apresenta as ideias em uma sequência lógica	1,00
14. Aborda o tema atual	1,00
15. Tem um tamanho de texto adequado	1,00
**RELEVÂNCIA: significância, impacto, motivação e interesse**	
16. Estimula o aprendizado	1,00
17. Contribui para o conhecimento na área	1,00
18. Desperta interesse pelo tema	1,00
Total	0,99

* *IVC - Índice de Validade de Conteúdo.*

*Fonte: Adaptado de Leite et al. (2018).*

## DISCUSSÃO

A validação do conteúdo de uma tecnologia de ensino e aprendizagem assume um papel pedagógico central em um cenário de múltiplos meios de divulgação de informações, nos quais muitas vezes circulam conteúdos sem respaldo científico, podendo induzir o indivíduo ao erro. Essa situação é preocupante para a formação inicial e continuada profissionais, principalmente na área da saúde, em que decisões baseadas em informações inadequadas podem comprometer a segurança das pessoas^(2,16)^.

O IVCES, utilizado neste estudo, foi desenvolvido para auxiliar na validação de materiais educativos destinados a qualquer público-alvo, preservando os aspectos didáticos-pedagógicos. Seus itens foram elaborados com base nos princípios do design instrucional, que orientam sistematicamente o planejamento e a construção de materiais para a educação nas diversas modalidades de ensino (presencial, semipresencial e à distância). Por essas características, o instrumento se mostra adequado para validação do protótipo desenvolvido nesta pesquisa^(25)^. De forma semelhante com o presente estudo, uma pesquisa que validou uma tecnologia sobre sepse em adultos usou o IVCES para avaliar o conteúdo e empregou formulário eletrônico via *Google Forms* ^(29)^.

Raros são os estudos encontrados sobre validação de conteúdo de tecnologias sobre sepse voltados à educação. Entre esses poucos, Limeira *et al.* (2023) validaram um aplicativo sobre sepse em adultos, destinado à educação da população geral^(29)^. Já Evans *et al.* (2015) e Adhikari *et al.* (2021) desenvolveram um jogo sobre sepse em adultos, mas não relataram a validação de conteúdo; em vez disso, apenas avaliaram o efeito do jogo no conhecimento de estudantes^(12,14)^.

Em uma busca simples na literatura, observa-se que a maioria dos estudos disponíveis não são voltados para educação dos profissionais apesar da crescente adoção do ensino à distância^(30)^. Moraes *et al.* (2022) valorizam a utilização de jogos educativos na educação continuada de profissionais e preveem a necessidade de mais jogos direcionados à educação sobre infecção hospitalar^(31)^. Marotta e Mello (2019) referem que o ensino em sala de aula requer dinamismo e interatividade e que os professores precisam se adequar aos novos métodos de ensino e usá-los da melhor maneira possível; uma estratégia para isso é criando softwares específicos de educação conforme a área de interesse^(2)^.

Uma das principais regras para desenvolver um jogo de aprendizagem é que a meta de jogo e o objetivo de aprendizagem sejam claros. Pela avaliação dos juízes, pode-se dizer que o protótipo avaliado no presente estudo atendeu a essa normativa. Um jogo só pode ser considerado jogo sério quando está ligado a um objetivo concreto, ao mesmo tempo em que entretém o jogador; desse modo, é capaz de não só promover conhecimento, mas também de provocar mudanças de comportamento^(4,31,32)^.

Em um jogo sério, deve-se propor sempre o conhecimento respaldado por evidências científicas atualizadas, já que o material visa ser útil na vida acadêmica e profissional do jogador. Nesse sentido, as referências utilizadas neste protótipo foram as publicadas mais recentemente por organizações nacionais e internacionais^(19-21)^. Esse cuidado com a atualização das evidências também é observado em outros estudos: Limeira et al., em seu aplicativo sobre sepse em adultos, utilizaram referências semelhantes às deste estudo, assim como Evans et al. (2015), ambos em consonância com o período de criação e publicação de cada tecnologia^(14,29)^.

O protótipo propôs um esquema de jogo em cinco fases. Esse plano de ensino gradativo - e com sequência lógica semelhante ao passo a passo do atendimento à sepse pediátrica - favorece o aprendizado e qualifica o jogador para atuar em casos reais^(4,33)^.

A linguagem utilizada buscou atender tanto estudantes quanto profissionais, sem perder a taxonomia própria da área da saúde, possibilitando manter o jogador atento por meio de um jogo atrativo^(25,33)^.

No tocante ao IVC, outros estudos de validação de tecnologia também utilizaram essa medida na validação de tecnologias educativas, visto que é sugerida para avaliar o conteúdo de materiais, sobretudo educativos^(29,34)^. Notou-se um valor de IVC superior ou semelhante ao de outras pesquisas. Isso pode decorrer do método utilizado para a avaliação do protótipo seguindo o *design thinking*. A realização da primeira análise, conduzida item a item nas telas do protótipo-piloto, permitiu maior detalhamento da avaliação. A segunda análise, por sua vez, contemplou a avaliação das fases e o uso de um instrumento específico para validação de conteúdo educativo, conferindo caráter conclusivo ao processo. Uma vantagem observada foi que os juízes eram os mesmos que haviam tido contato prévio com o piloto, o que lhes proporcionou maior familiaridade com o material e tornou a avaliação do conteúdo mais fácil e rápida^(18,34,35)^.

Além disso, este protótipo buscou seguir as recomendações de criação de jogo de aprendizagem, as quais abarcam a grande maioria dos itens avaliados pelo instrumento utilizado. Tais orientações incluem deixar claro, desde o início do jogo, o objetivo de aprendizagem; utilizar linguagem simples e objetiva; empregar recursos visuais e terminologia próprios da área da saúde; e abordar o tema a partir de suas prioridades, entre elas a identificação dos sinais e sintomas de sepse em crianças, as condutas imediatas após a suspeita, a identificação das causas e a prevenção das IRAS^(4,25,33,36)^.

Nascimento (2021), ao validar um jogo sobre prevenção de infecção cirúrgica, utilizou a estratégia de roteiro para a validação do conteúdo. O autor preferiu validá-lo já em sua forma dentro do jogo e, assim, facilitar o entendimento dos juízes sobre como o conteúdo seria ofertado^(35)^.

Ressalta-se a importância de que a criação do protótipo e a validação do conteúdo sejam feitas dentro do processo de criação do software, uma vez que o protótipo serve de guia para os juízes, evitando dispêndio de tempo e até custos extras com o material^(22)^.

### Limitações do Estudo

Destaca-se como limitações do estudo o caráter não probabilístico e intencional da amostra de juízes, bem como o fato de o conteúdo ter sido validado para aplicação a estudantes e profissionais de saúde vinculados ao local de estudo. Dessa forma, não foram considerados possíveis aspectos culturais de outras regiões que poderão utilizar o material, ainda que o conteúdo tenha sido elaborado com base em consensos nacionais e internacionais.

### Contribuições para a Área

A validação realizada por este estudo contribui para divulgação de conteúdo fiel e responsável à estudantes e profissionais da saúde sobre a sepse pediátrica.

## CONCLUSÕES

O IVC total igual a 0,99 indica que o conteúdo deste jogo contempla os objetivos de aprendizagem, tem estrutura e apresentação favoráveis ao aprendizado e mostra-se relevante para o ensino na atualidade. Agora que o conteúdo está validado, torna-se viável avançar para a etapa de validação da usabilidade mediante a aplicação do jogo ao público-alvo, visando verificar sua eficácia como estratégia de ensino e aprendizagem sobre sepse pediátrica.

## Data Availability

Os dados de pesquisa estão disponíveis no corpo do artigo.
